# Association between Voluntary/Involuntary Job Loss and the Development of Stroke or Cardiovascular Disease: A Prospective Study of Middle-Aged to Older Workers in a Rapidly Developing Asian Country

**DOI:** 10.1371/journal.pone.0113495

**Published:** 2014-11-19

**Authors:** Mo-Yeol Kang, Hyoung-Ryoul Kim

**Affiliations:** 1 Occupational Safety and Health Research Institute, Korea Occupational Safety and Health Agency, Ulsan, South Korea; 2 Department of Preventive Medicine, Seoul National University College of Medicine, Seoul, South Korea; 3 Department of Occupational and Environmental Medicine, College of Medicine, the Catholic University of Korea, Seoul, South Korea; University of Tennessee, United States of America

## Abstract

**Background:**

The aim of this research was to investigate the association between job loss and the development of stroke or cardiovascular disease among middle-aged to older individuals in Korea. We also examined how this relationship was modified by gender and the nature of the job loss.

**Methods:**

This study used samples from the first- to fourth-wave datasets from the Korean Longitudinal Study of Aging (KLoSA), which were collected in 2006, 2008, 2010, and 2012. The study collected data from a total of 10,254 subjects aged ≥45 years at baseline. After applying exclusion criteria, the final sample size for analysis consisted of 4,000 individuals. Information about employment status, development of stroke or cardiovascular disease, and covariates (age, income level, and behavioral factors) was obtained. Cox proportional hazards models were used to evaluate the association between voluntary/involuntary job loss and the development of stroke or cardiovascular disease. We performed these analyses separately according to disease, gender, and the nature of the job loss.

**Results:**

Involuntary job loss significantly increased the risk of stroke or cardiovascular disease among males (adjusted hazard ratio [HR] = 3.560, 95% confidence interval [CI] = 2.055–6.168). Voluntary retirement also increased the risk of cardiovascular disease or stroke among males (adjusted HR = 2.879, 95% CI = 1.533–5.409). Job loss was more closely associated with stroke than with cardiovascular disease (stroke, adjusted HR = 6.208, 95% CI = 2.417–15.943; cardiovascular disease, adjusted HR = 2.768, 95% CI = 1.402–5.465).

**Conclusion:**

Our findings suggest that both voluntary retirement and involuntary job loss increase the risk for stroke or cardiovascular disease in middle-aged to older individuals, especially males.

## Introduction

Unemployment is a major social problem worldwide, with serious economic and health consequences for affected individuals. Job loss is an inevitable feature of the current market economy, and individuals may experience serious health consequences after job loss. For adults, unemployment and job loss are two of the most stressful life events, which can lead to decreased social status, disrupted family and social roles [1], financial strain [2], and loss of self-esteem [3].

Understanding the health consequences of unemployment is important for a complete understanding of the effects of economic downturns. Unemployment is common during these downturns, especially among older workers. The relationship between unemployment and poor health has been well researched, and findings indicate that there are higher prevalence rates of physical and mental diseases and higher mortality rates among unemployed individuals [4]. Involuntary job loss is a stressful life event that has significant negative health consequences, especially among older workers [5]–[8]. The results of one study suggest that retirement and poor mental health are related, even when retirement is voluntary and pre-planned [9]. However, other investigators found that the effects of voluntary retirement are positive, or at worst neutral, abut that involuntary retirement has negative effects [10]. Despite common beliefs that retirement, in and of itself, can have negative health effects, the adverse effects of retirement on physical health have not been satisfactorily described [11].

A number of studies have examined the health effects of unemployment on the risk of cardiovascular disease. Some ecological study investigators reported significant associations between the unemployment rate and cardiovascular [12] and cerebrovascular mortality [13]. Authors of prospective studies have reported increased prevalence rates of hypertension [14], coronary heart disease [15]–[18], and cardiovascular and cerebrovascular mortality among unemployed compared with employed workers [19], [20]. However, most of these studies assessed relatively small and younger populations or were conducted in developed Western countries. Few studies have investigated gender differences in cardiovascular risk due to unemployment or have compared differences between involuntary job loss and voluntary retirement. We considered the limitations and challenges of the broader body of available evidence and used a large sample of representative data from the Korean population. Korea is one of the most rapidly developing Asian countries. A positive relationship between unemployment and cardiovascular risk or stroke has been well demonstrated [6], [15]–[19]. However, reverse causality may have contributed to the results because workers with poor health may have disadvantages in the labor market and an increased risk of job loss (i.e., the health selection hypothesis). To assess causal relationships in more detail, we used a prospective study design and excluded individuals who retired because of health problems and individuals with cerebrovascular disease or heart problems at baseline.

The aim of this research was to determine the association between job loss and the development of stroke or cardiovascular disease among middle-aged to older individuals. A middle-aged to older worker's job loss may have deleterious effects on the cardio- and cerebrovascular systems. Identifying the characteristics associated with this effect (e.g., gender differences, differences between involuntary job loss and voluntary retirement) would be helpful for the development of strategies to prevent stroke and cardiovascular disease. Therefore, we also examined how the relationship between job loss and the development of stroke or cardiovascular disease is modified by gender and the nature of the job loss.

## Materials and Methods

### Data collection and participants

This study used a sample derived from the first- to fourth-wave datasets of the Korean Longitudinal Study of Aging (KLoSA), conducted by the Korea Labor Institute (Seoul) and Korea Employment Institute Information Service (Seoul). The surveys were conducted in 2006, 2008, 2010, and 2012. The original KLoSA study population was comprised of South Korean adults, aged 45 years or older, who resided in one of 15 large administrative areas. In 2006, 15 major cities and provinces were selected using stratification, and 10,000 households were randomly selected from these populations. Successful interviews were performed in 6,171 of the 10,000 selected households. A total of 10,254 subjects were surveyed. These subjects were followed up on a biennial basis until 2012.

The participants were interviewed using the Computer-Assisted Personal Interviewing method. The interviewers instructed the subjects to read the questions and then input the answers without assistance. The first set of interviews was conducted from August through December 2006, the second set from July through November 2008, the third set from October through December 2010, and the fourth set from July through December 2012. The second survey in 2008 followed up with 8,688 subjects, who represented 86.9% of the original panel; the third survey in 2010, included 7,920 subjects (77.2% of the original panel); and the fourth survey in 2012 included 7,486 subjects (73.0% of the original panel).

The KLoSA is a national public database (http://www.kli.re.kr/klosa/en/about/introduce.jsp) that includes an identification number for each participant; however, the number is not associated with any personal identifying information. The data collection system and database were designed to protect subject confidentiality. Participants were required to read and sign an agreement form before participating in the KLoSA study and to consent that their data could be used in future scientific research.

**Figure 1:**
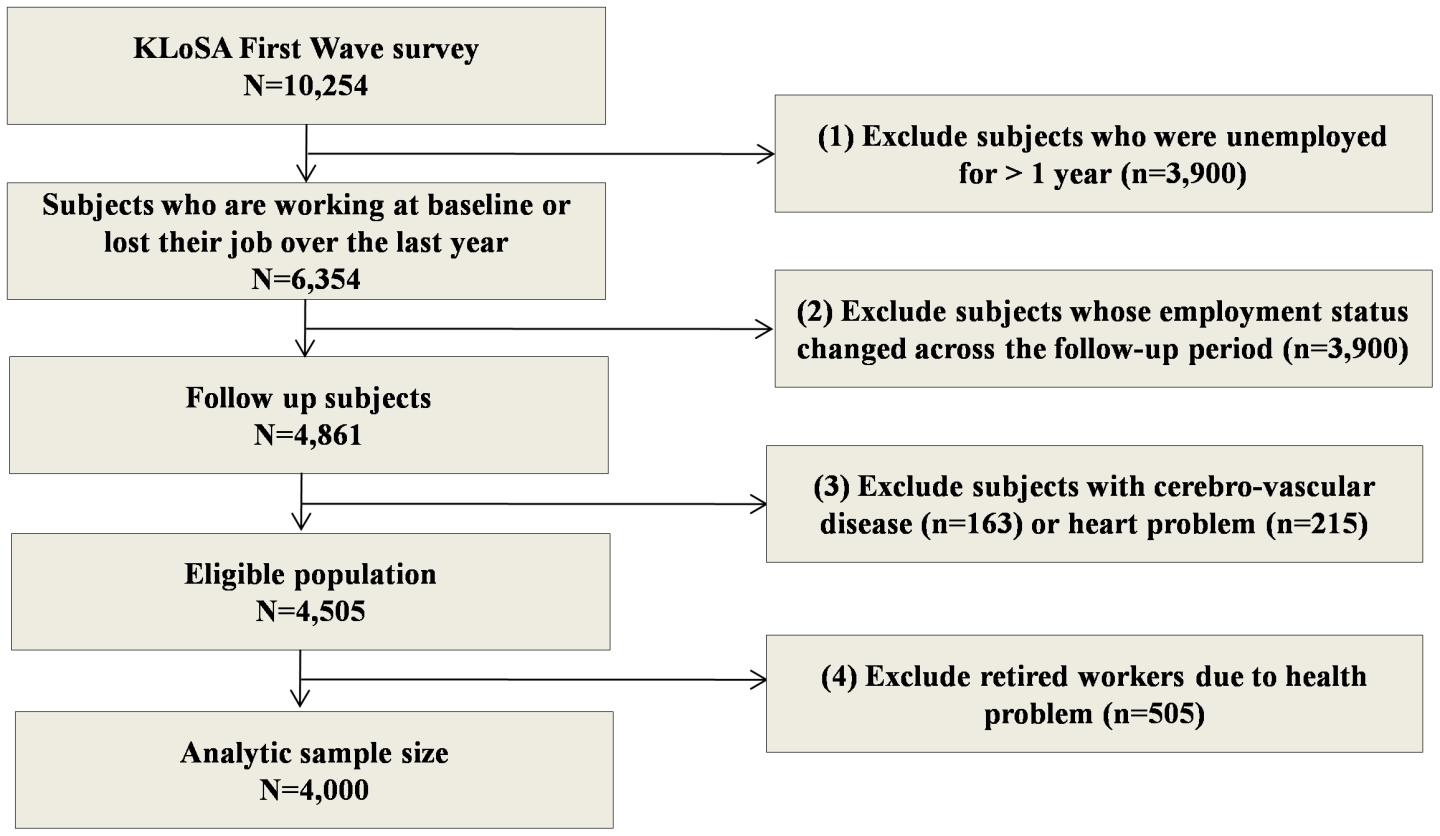
Schematic diagram of the study population.

We used the following exclusion criteria: (1) We excluded 3,900 subjects who were unemployed for >1 year. Only subjects who were employed at baseline and had lost their job during the year prior to the first survey were selected from the 10,254 subjects included in the first dataset (n = 6,354). (2) After that time, a total of 1,493 subjects who experienced a change in employment status across the follow-up periods were excluded (n = 4,861). (3) We also excluded 356 subjects with cerebrovascular or cardiac disease at baseline (n = 4,505). (4) After the elimination of 505 workers who retired due to health problems, the final sample size for analysis consisted of 4,000 subjects (Figure 1).

### Study variables and measurements

We defined the victims of involuntary job loss as those individuals who retired before their scheduled or regular retirement age due to business closure, layoff, or family problems.

New cases of stroke and cardiovascular disease were defined as those individuals who reported physician-diagnosed stroke or cardiovascular disease on a follow-up questionnaire. The questions were, “Since the last interview date, has a doctor told you that you had a heart attack, coronary heart disease, angina, congestive heart failure, or other heart problems?”, and “Have you been diagnosed with stroke since the last interview date?”. The subsequent questions “In what year and month was your stroke first diagnosed?” and “In what year and month was your cardiovascular disease first diagnosed?” determined the diagnosis dates of stroke and cardiovascular disease during the follow-up period. In the case of a subject's death, the cause of death and the disease diagnosis were confirmed by interviewing one or more of the subject's family members. The date of diagnosis was also asked of all subjects affected by those diseases or their family members. The follow-up period was calculated as the difference between the date of the first survey and the date of diagnosis. In undiagnosed subjects, the follow-up period was calculated from the date of the first survey to the date of the fourth survey. If undiagnosed subjects were lost to follow-up across the second to fourth set of interviews, their follow-up period was calculated as the difference between the date of the first survey and the date of the final survey that they completed.

The KLoSA survey included questions about a wide array of characteristics. We used age, gender, income level, chronic disease diagnosis, and health behavior variables. Questions like “Has a doctor ever told you that you have high blood pressure or hypertension?” and “Has a doctor ever told you that you have diabetes?” in the first survey were used to get information about subjects' medical history of hypertension or diabetes. Income level among individuals in the study population was stratified into tertiles based on annual household income rank, with the first tertile representing the lowest level. Information about lifestyle behaviors (e.g., cigarette smoking, alcohol consumption, regular exercise, and body mass index [BMI]) was collected during the first survey. Physical activity was categorized as regularly performed or not regularly performed. Regular exercise was defined as exercise more than twice per week, with each session lasting at least 30 minutes. Smoking habit was categorized as current smokers, ex-smokers, and nonsmokers. We defined heavy drinking as consuming at least 5 glasses of alcohol beverage in a single occasion at least 5 days in the past 30 days according to the definition provided by the Substance Abuse and Mental Health Services Administration. BMI was calculated using height and weight variables as weight/height^2^ (kg/m^2^); a BMI of ≥25.0 was regarded as obese, and a 25≥BMI >23 was regarded as overweight according to the World Health Organization Asia-Pacific guidelines [21].

### Statistical analyses

We first compared the descriptive characteristics between subjects followed up until fourth-wave and those who failed to follow up. The general and clinical characteristics of the study population were summarized. We calculated the frequencies of the baseline characteristics of the participants and compared them to each categorized variable for analysis. Cox proportional hazards models were used to evaluate the association between voluntary/involuntary job loss and the development of stroke or cardiovascular disease. Covariates associated with the development of stroke or cardiovascular disease were determined using stepwise selection of Cox regression analysis, which identified age, hypertension, diabetes, and employment status as significant explanatory variables. Then, an advanced model was built based on clinical significance, including behavioral factors such as smoking, heavy drinking, regular exercise, and BMI, which are known as potential risk factors for stroke and cardiovascular disease. In the final model, we also adjusted for income level. Even though it was not statistically significant, socioeconomic status might be closely associated with disease development. Therefore, we employed four models: Model 1 (adjusted for age), Model 2 (adjusted for age, hypertension, and diabetes), Model 3 (adjusted for age, hypertension, diabetes, and behavioral factors including smoking, heavy drinking, regular exercise, and BMI), and Model 4 (adjusted for age, hypertension, diabetes, behavioral factors, and income level). Two approaches were used to assess the validity of the proportional hazards assumption. First, we examined graphs of the log-minus-log-survival functions and found that the plot had parallel lines. Second, we used a time-dependent covariate to confirm proportionality and found that the time-dependent covariate was not statistically significant (p-value  = 0.6423), suggesting that the hazard is reasonably constant over time. Separate analyses were performed for each outcome of interest (stroke only and cardiovascular disease only). Because the results could be modified by gender, we also performed an analysis including both genders and investigated effect modification by assessing the interaction term involving gender. Statistical analyses were performed using SAS (Version 9.22, SAS Institute, Cary, NC, USA) statistical software. A two-tailed p-value <0.05 was considered to indicate statistical significance.

## Results

We summarized the results for the final survey for respondents and non-respondents in Table 1. Respondents and non-respondents appeared similar with respect to the distributions of general characteristics. However, the distributions of employment status and smoking habit were significantly different (Table 1).

**Table 1:** General characteristics of respondents compared with those of non-respondents at wave 4.

Characteristics	Respondents		Non-Respondents		P-value
	n	%	n	%	
**Demographics**					
Gender					0.2785
Male	1,868	64.73	736	66.07	
Female	1,018	35.27	378	33.93	
Age					
45–54	1,315	45.56	552	49.55	
55–64	779	26.99	258	23.16	0.1618
≥65	792	27.44	304	27.29	
Income level					
Low	930	32.22	407	36.54	
Middle	1,013	35.10	317	28.46	0.4929
High	943	32.67	390	35.10	
**Employment status**					
Still employed	2,040	70.69	709	63.64	
Voluntary retirement	370	12.82	187	33.57	0.0002
Involuntary job loss	476	16.49	218	19.57	
**Chronic disease**					
Hypertension					
No	2,278	78.93	905	81.24	0.1049
Yes	608	21.07	209	18.76	
Diabetes					
No	2,617	90.68	1,016	91.20	0.6070
Yes	269	9.32	98	8.80	
**Health behaviors**					
Smoking					
Non-smoker	1,710	59.27	637	57.18	
Ex-smoker	407	10.18	129	11.58	0.0303
Current smoker	768	26.62	348	31.24	
Heavy drinking					
No	2,430	85.41	987	85.45	0.9731
Yes	415	14.59	168	14.55	
Regular exercise					
Yes	564	19.54	212	19.03	0.7135
No	2,322	80.46	902	80.97	
BMI					
Normal or underweight	1,301	45.08	514	46.14	
Overweight	889	30.80	346	31.06	0.4015
Obese	696	24.12	254	22.80	
**Total**	2,886	72.15	1,114	27.85	

The mean age (and corresponding standard deviation [SD]) of the analyzed subjects was 57.82±10.66 years, and two-thirds of them were male. The proportion of smokers was 40.91% among male subjects, but female smokers were very rare (Table 2). The proportion of heavy drinkers in the study population was 14.59%, and most of them were male (male, 565; female, 18). About one-quarter of the study population was obese, and more than one-fifth of subjects did not exercise regularly. The frequency of hypertension was around 20% in both male and female subjects, and 10% had diabetes. The other descriptive characteristics of the study population are presented in Table 2. Descriptive statistics subdivided by employment status are presented in Table 3.

**Table 2:** Baseline general characteristics of the study population.

	Male		Female	
Characteristics	n	%	n	%
**Demographics**				
Age				
45–54	1,129	43.36	738	52.87
55–64	678	26.04	359	25.72
≥65	797	30.61	299	21.42
Income level				
Low	816	31.34	521	37.32
Middle	880	33.79	450	32.23
High	908	34.87	425	30.44
**Employment status**				
Still employed	1,854	71.20	895	64.11
Voluntary retirement	277	10.64	280	20.06
Involuntary job loss	473	18.16	221	15.83
**Chronic disease**				
Hypertension				
No	2,065	79.30	1,118	80.09
Yes	539	20.70	278	19.91
Diabetes				
No	2,344	90.02	1,289	92.34
Yes	260	9.98	107	7.66
**Health behaviors**				
Smoking				
Non-smoker	1,010	38.80	1,337	95.77
Ex-smoker	528	20.28	8	0.57
Current smoker	1,065	40.91	51	3.65
Heavy drinking				
No	2,039	78.30	1,378	98.71
Yes	565	21.70	18	1.29
Regular exercise				
Yes	546	20.97	230	16.48
No	2,058	79.03	1,166	83.52
BMI				
Normal or underweight	1,132	43.47	683	48.93
Overweight	878	33.72	357	25.57
Obese	594	22.81	356	25.50
**Total**	2,604	100	1,396	100

BMI, body mass index.

**Table 3:** General characteristics of the study population divided by employment status at baseline.

	Total		Employment status					
			Employed		Voluntary retirement		Involuntary Job loss	
Characteristics	N	%	n	%	n	%	n	%
**Demographics**								
Gender								
Male	2,604	65.10	1,854	67.44	277	49.73	473	68.16
Female	1,396	34.90	895	32.56	280	50.27	221	31.84
Age								
45–54	1,867	46.68	1,663	60.49	131	23.52	73	10.52
55–64	1,037	25.93	726	26.41	132	23.70	179	25.79
≥65	1,096	27.40	360	13.10	294	52.78	442	63.69
Income level								
Low	1,337	33.43	666	24.23	269	48.29	402	57.93
Middle	1,330	33.25	997	36.27	149	26.75	184	26.51
High	1,333	33.33	1,086	39.51	139	24.96	108	15.56
**Chronic disease**								
Hypertension								
No	3,183	79.58	2,319	84.36	387	69.48	477	68.73
Yes	817	20.43	430	15.64	170	30.52	217	31.27
Diabetes								
No	3,633	90.83	2,550	92.76	495	88.87	588	84.73
Yes	367	9.18	199	7.24	62	11.13	106	15.27
**Health behaviors**								
Smoking								
Non-smoker	2,347	58.69	1,570	57.13	397	71.27	380	54.76
Ex-smoker	536	13.40	329	11.97	67	12.03	140	20.17
Current smoker	1,116	27.91	849	30.90	93	16.70	174	25.07
Heavy drinking								
No	3,417	85.43	2,289	83.27	517	92.82	611	88.04
Yes	583	14.58	460	16.73	40	7.18	83	11.96
Regular exercise								
Yes	776	19.40	383	13.93	162	29.08	231	33.29
No	3,224	80.60	2,366	86.07	395	70.92	463	66.71
BMI								
Normal or underweight	1,815	45.38	1,209	43.98	266	47.76	340	48.99
Overweight	1,235	30.88	897	32.63	157	28.19	181	26.08
Obese	950	23.75	643	23.39	134	24.06	173	24.93
Total	4,000	100	2,748	68.72	557	13.93	694	17.35

The results of the survival analyses are presented in Tables 4 and 5, for male and female subjects, respectively. For both outcomes (cardiovascular disease and stroke), unadjusted and adjusted hazard ratios (with associated 95% confidence intervals [CIs]) are presented using the Cox proportional hazard model results and the employed group as a reference. The best models for our analysis were determined using stepwise Cox regression analysis. The base model for prediction of stroke and cardiovascular disease included participants' age, history of diabetes and hypertension, and employment status (DF = 7, p-value <0.0001); extended models added variables addressing effects of gender, income level, smoking and drinking habits, regular exercise, and BMI. The likelihood ratio chi-square statistic was used to compare the fitted model to a model without covariates, and a -2 Log Likelihood statistic showed overall significance of the set of covariates included in the final model (DF = 14, p-value <0.0001).

**Table 4:** Risk of cardiovascular disease and stroke associated with voluntary retirement and involuntary job loss in males.

		Stroke		Cardiovascular disease		Total	
Events	Employed	14/1,854	0.76%	35/1,854	1.89%	45/1,854	2.43%
	Voluntary retirement	13/277	4.69%	13/277	4.69%	26/277	10.64%
	Involuntary job loss	32/473	6.77%	36/473	7.61%	64/473	13.53%
	Total	59/2,604	2.27%	84/2,604	3.23%	135/2,604	5.18%
		**HR**	**95% CI**	**HR**	**95% CI**	**HR**	**95% CI**
Model 1	Employed	1	reference	1	reference	1	reference
	Voluntary retirement	5.807	2.079–16.219	2.302	1.044–5.077	3.348	1.803–6.216
	Involuntary job loss	8.272	3.387–20.201	3.338	1.754–6.351	4.389	2.611–7.377
Model 2	Employed	1	reference	1	reference	1	reference
	Voluntary retirement	5.390	1.943–14.950	2.177	0.999–4.745	3.124	1.695–5.759
	Involuntary job loss	7.286	2.972–17.863	2.870	1.512–5.446	3.823	2.273–6.429
Model 3	Employed	1	reference	1	reference	1	reference
	Voluntary retirement	4.926	1.741–13.937	2.116	0.959–4.671	2.944	1.577–5.496
	Involuntary job loss	6.916	2.743–17.436	2.822	1.452–5.484	3.668	2.139–6.290
Model 4	Employed	1	reference	1	reference	1	reference
	Voluntary retirement	4.494	1.573–12.835	2.097	0.942–4.669	2.879	1.533–5.409
	Involuntary job loss	6.208	2.417–15.943	2.768	1.402–5.465	3.560	2.055–6.168

Model 1 is adjusted for age. Model 2 is adjusted for age, hypertension, and diabetes. Model 3 is adjusted for age, hypertension, diabetes, and behavioral factors including smoking, heavy drinking, regular exercise, and BMI. Model 4 is adjusted for age, hypertension, diabetes, behavioral factors, and income level.

CI, confidence interval; HR, hazard ratio.

**Table 5:** Risk of cardiovascular disease and stroke associated with voluntary retirement and involuntary job loss in females.

		Stroke		Cardiovascular disease		Total	
Events	Employed	7/895	0.78%	16/895	1.79%	23/895	2.57%
	Voluntary retirement	8/280	2.86%	11/280	3.93%	19/280	6.79%
	Involuntary job loss	6/221	2.71%	10/221	4.52%	16/221	7.24%
	Total	21/1,396	1.50%	37/1,396	2.65%	58/1,396	4.15%
		**HR**	**95% CI**	**HR**	**95% CI**	**HR**	**95% CI**
Model 1	Employed	1	reference	1	reference	1	reference
	Voluntary retirement	2.844	0.888–9.108	1.495	0.576–3.879	1.715	0.833–3.533
	Involuntary job loss	1.834	0.466–7.217	1.820	0.674–4.918	1.438	0.637–3.246
Model 2	Employed	1	reference	1	reference	1	reference
	Voluntary retirement	2.811	0.870–9.085	1.450	0.555–3.788	1.606	0.772–3.345
	Involuntary job loss	1.819	0.467–7.085	1.791	0.665–4.824	1.435	0.639–3.221
Model 3	Employed	1	reference	1	reference	1	reference
	Voluntary retirement	2.850	0.859–9.450	1.750	0.651–4.704	2.357	1.155–4.810
	Involuntary job loss	1.559	0.373–6.516	1.968	0.707–5.482	1.918	0.6866–4.250
Model 4	Employed	1	reference	1	reference	1	reference
	Voluntary retirement	2.980	0.889–9.985	1.783	0.661–4.809	2.410	1.177–4.934
	Involuntary job loss	1.598	0.381–6.704	1.876	0.670–5.247	1.864	0.839–4.140

Model 1 is adjusted for age. Model 2 is adjusted for age, hypertension, and diabetes. Model 3 is adjusted for age, hypertension, diabetes, and behavioral factors including smoking, heavy drinking, regular exercise, and BMI. Model 4 is adjusted for age, hypertension, diabetes, behavioral factors, and income level.

CI, confidence interval; HR, hazard ratio.

We found that involuntary job loss significantly increased the risk of stroke or cardiovascular disease among male but not female subjects (male, adjusted HR = 3.560, 95% CI = 2.055–6.168, Table 4; female, adjusted HR = 1.864, 95% CI = 0.839–4.140, Table 5). Similarly, the association between voluntary retirement and development of cardiovascular disease or stroke was significant among both male and female subjects, but the relationship was stronger among male subjects (male, adjusted HR = 2.879, 95% CI = 1.533–5.409; female, adjusted HR = 2.410, 95% CI = 1.177–4.934). One of most interesting findings was that job loss was more closely associated with stroke than with cardiovascular disease in males (stroke, adjusted HR = 6.208, 95% CI = 2.417–15.943; cardiovascular disease, adjusted HR = 2.768, 95% CI = 1.402–5.465) (Table 4). The results of analysis including both male and female subjects are summarized in Table 6. This analysis showed that voluntary/involuntary job loss was more closely associated with the development of stroke or cardiovascular disease in males than in females, but the interaction term was not statistically significant (p-interaction  = 0.1318). The risk of both stroke and cardiovascular disease for male subjects who retired voluntarily was almost 3-fold higher compared with individuals who continued to work, and it was more than 3.5 times higher for those who experienced involuntary job loss. HRs for the covariates that were adjusted in the final model are also summarized in Table 7.

**Table 6:** Risk of cardiovascular disease and stroke associated with voluntary retirement and involuntary job loss.

		Stroke		Cardiovascular disease		Total	
Events	Employed	21/2,749	0.76%	51/2,749	1.86%	68/2,749	2.47%
	Voluntary retirement	21/557	3.77%	24/557	4.31%	45/557	8.08%
	Involuntary job loss	38/694	5.48%	46/694	6.63%	80/694	11.53%
	total	80/2,604	2.00%	121/4,000	3.94%	193/4,000	4.83%
		**HR**	**95% CI**	**HR**	**95% CI**	**HR**	**95% CI**
Model 1	Employed	1	reference	1	reference	1	reference
	Voluntary retirement	4.343	2.012–9.374	1.923	1.050–3.522	2.728	1.726–4.312
	Involuntary job loss	5.284	2.594–10.761	2.751	1.627–4.651	3.300	2.179–5.000
Model 2	Employed	1	reference	1	reference	1	reference
	Voluntary retirement	4.187	1.940–9.038	1.855	1.013–3.395	2.633	1.666–4.162
	Involuntary job loss	4.892	2.401–9.970	2.483	1.470–4.194	3.035	2.003–4.597
Model 3	Employed	1	reference	1	reference	1	reference
	Voluntary retirement	3.966	1.806–8.707	1.893	1.025–3.498	2.635	1.652–4.202
	Involuntary job loss	4.785	2.290–10.000	2.567	1.494–4.409	3.092	2.011–4.753
Model 4	Employed	1	reference	1	reference	1	reference
	Voluntary retirement	3.723	1.691–8.199	1.830	0.987–3.392	2.595	1.595–4.074
	Involuntary job loss	4.415	2.088–9.336	2.444	1.407–4.245	2.955	1.908–4.577

Model 1 is adjusted for age and gender. Model 2 is adjusted for age, gender, hypertension, and diabetes. Model 3 is adjusted for age, gender, hypertension, diabetes, and behavioral factors including smoking, heavy drinking, regular exercise, and BMI. Model 4 is adjusted for age, gender, hypertension, diabetes, behavioral factors, and income level.

CI, confidence interval; HR, hazard ratio.

**Table 7:** The results of Cox proportional hazard regression analysis at the final models.

	Stroke		Cardiovascular disease		Total	
	HR	95% CI	HR	95% CI	HR	95% CI
Demographics						
Age						
45–54	1	reference	1	reference	1	reference
55–64	2.113	0.869–5.140	1.485	0.829–2.661	1.628	1.009–2.626
≥65	2.578	1.029–6.461	1.779	0.951–3.328	2.012	1.213–3.337
Gender (Male)	1.269	0.651–2.476	1.247	0.732–2.122	1.090	0.720–1.650
Income level						
Low	1.457	0.700–3.034	1.160	0.667–2.017	1.289	0.825–2.012
Middle	0.984	0.447–2.166	0.934	0.533–1.639	1.054	0.669–1.661
High	1	reference	1	reference	1	reference
Employment status						
Still employed	1	reference	1	reference	1	reference
Voluntary retirement	3.723	1.691–8.199	1.830	0.987–3.392	2.595	1.595–4.074
Involuntary job loss	4.415	2.088–9.336	2.444	1.407–4.245	2.955	1.908–4.577
Chronic disease						
Hypertension (Yes)	1.513	0.886–2.584	1.494	0.966–2.309	1.418	1.008–1.995
Diabetes (Yes)	1.723	0.914–3.248	2.256	1.393–3.656	1.975	1.339–2.914
Health behaviors						
Smoking						
Non-smoker	1	reference	1	reference	1	reference
Ex-smoker	0.571	0.238–1.37	1.069	0.589–1.942	0.855	0.518–1.412
Current smoker	1.684	0.906–3.132	1.341	0.797–2.255	1.519	1.013–2.277
Heavy drinking (Yes)	1.126	0.520–2.440	1.248	0.679–2.292	1.046	0.653–1.677
Regular exercise (Yes)	0.786	0.448–1.377	1.122	0.688–1.830	0.962	0.666–1.391
BMI						
Normal or underweight	1	reference	1	reference	1	reference
Overweight	1.087	0.557–2.119	1.126	0.681–1.860	1.078	0.721–1.611
Obese	1.550	0.867–2.771	1.142	0.714–1.824	1.269	0.882–1.826

## Discussion

Middle-aged to older male workers who became unemployed during the 1 year prior to the first survey had an increased risk for stroke or cardiovascular disease during the 8-year follow-up period. This risk was present regardless of whether the change in employment status was voluntary or involuntary. Furthermore, involuntary job loss was more closely associated with the development of both stroke and cardiovascular disease than voluntary retirement.

Our findings are consistent with those of previous studies. The 6-, 10-, and 18-year follow-ups of the US Health and Retirement Study examined myocardial infarction (MI) and stroke following job loss (layoff or plant closure) in workers aged ≥50 years. Job loss was found to be associated with stroke and MI (stroke, adjusted HR = 2.64; 95% CI = 1.01–6.94; MI, adjusted HR = 1.89, 95% CI = 0.91–3.9) [6], [22]. The results of another study of a Swedish military conscription cohort revealed that unemployment ≥90 days elevated the risk of coronary heart disease during the 8-year follow-up (HR = 1.24, 95% CI = 1.04–1.48) [18]. In another Swedish study based on a registry linkage of 3.4 million individuals, unemployment was found to be significantly associated with mortality from circulatory diseases, including ischemic heart disease (HR = 1.17) and stroke (HR = 1.44), during the 6-year follow-up period [20]. However, a large French occupational cohort (the GAZEL study) showed that retirement did not change the risks of coronary heart disease or stroke [23]. Cultural differences between countries and work environment might have contributed to the inconsistent findings [16].

The results described above are from studies of populations in Western countries. Our findings indicate that the risks of stroke and cardiovascular disease associated with unemployment were much higher in Korea, which is one of the most rapidly developing Asian countries. According to statistics from the Organization for Economic Cooperation and Development countries, Korea is distinguished by low unemployment rates among adults ≥45 years old. In 2012, unemployment rates were 1.9% for individuals 45 to 54 years of age, 2.5% for those 55 to 64 years, and 2.1% for individuals ≥65 years. These rates are lower than the mean levels reported for Organization for Economic Co-operation and Development countries (6.0% [45 to 54 years], 5.7% [55 to 64 years], and 3.5% [≥65 years]) [24]. The reason for this difference may be related to the low coverage rate of old-age pensions and social security systems in Korea [25]. Only 28% of all elderly people in Korea receive a basic old-age pension. Moreover, the proportion of expenditure for medical services is relatively high (almost 50% of total income) in this age group. Therefore, an older worker in Korea who leaves their job is more likely to suffer from both decreasing income and increasing health-related payments [26]. These individuals may experience considerably greater health care difficulties compared with individuals in the Western countries with better social welfare systems.

Our findings indicate that compared with voluntary retirement, involuntary job loss had more serious health effects among middle-aged to older male workers. This finding is consistent with the results of a report by Gallo et al., who found that the risk of subsequent stroke was associated with involuntary job loss (adjusted HR = 2.64; 95% CI = 1.01–6.94). However, that study did not compare the differences between involuntary job loss and voluntary retirement. Involuntary job loss later in life is a stressful event that has significant adverse health and behavioral consequences, such as poorer physical function [7], increased alcohol consumption [8] and increased risk of hospitalization due to alcohol-related disease [19], [27], increased smoking intensity and a greater tendency to relapse [5], and a greater risk of eventual MI and stroke [6]. Unexpected job loss in later life may have negative effects for older workers that extend beyond economic problems. Unexpected or unwanted job loss creates stress by disrupting extensive, careful planning and decision-making processes based on future expectations [28]. Moreover, the loss of control related to involuntary job loss may leave older workers with a sense that the circumstances affecting their lives are beyond their control [29]. For many older workers, involuntary job loss can cause substantial loss of income, the dissolution of close social interactions [30], and the dishonor of unemployment, which may act together or alone to create stress.

In our study, Model 3 was further adjusted for behavioral factors such as smoking, heavy drinking, BMI, and regular exercise, and Model 4 was adjusted for income level. Because these variables and employment status were surveyed at the same time, it is possible that these values were affected by employment. A previous study suggested that unemployment itself may affect individual health behaviors [31]. In our analysis, health behavioral factors were associated with employment status but not the risk of stroke or cardiovascular disease. On the other hand, an association between income level and risk of stroke or cardiovascular disease was statistically significant in univariate analysis, but the HR was decreased after adjusting for employment status (univariate, HR = 2.543, p-value <.0001; Adjusting for employment status, HR = 1.611, p-value  = 0.0266). These findings suggest that earning losses after job loss may be associated with a greater risk for cerebro-cardiovascular disease, but other factors caused by job loss also significantly affect the development of cerebro-cardiovascular disease. Further studies more focused on identifying causal pathways are encouraged to enhance our understanding of how unemployment increases the risk of cerebro-cardiovascular disease.

The other important finding of this study is that even voluntary retirement has large effects on the risks of stroke and cardiovascular disease. Voluntary retirement is usually a planned decision reached after ensuring financial stability. However, voluntary retirement is also associated with lifestyle changes, which could lead to distress [9]. Kim and Moen suggested that when late-midlife individuals transition into retirement, they experience a short-term post-retirement boost in morale or general satisfaction, but their long-term distress levels appear to increase [32]. Moreover, the low coverage rates of old-age pensions and social security systems in rapidly developing Asian countries might cause hardship for retirees to adapt themselves to new circumstances.

Another important finding of our study is that unemployment was more closely associated with the development of stroke or cardiovascular disease in males than in females, although the interaction between gender and employment status was not statistically significant. The results of a study of a single plant closing revealed that unemployment has a greater effect on depression in males compared with females [33]. Artacoz et al. also reported that unemployment had more significant effects on the mental health of males than females, and they proposed that the gender differences were related to family responsibilities and social class [34]. They suggested that gender differences in the health effects of unemployment might be due to the still existing influence of the male breadwinner–female homemaker model in older individuals. Working can itself contribute to the double burden of paid and domestic work for women. Cultural expectations about a woman's role in paid employment and unpaid work in the family may lead to lowered expectations of control and stability in work for women. Therefore, a woman may experience involuntary job loss or voluntary retirement less negatively and with fewer negative health consequences compared with men. Men tend to recognize job loss as social failure, whereas women tend to regard job loss as a chance to spend more time with their family [26]. Upon being advised to resign, women are more likely to give up their desire to work than men because they can shift their role from work to homemaker.

The finding that job loss is more closely associated with stroke than cardiovascular disease is noteworthy but is somewhat difficult to interpret. This finding was also observed in the 6-year follow-up study of the US Health and Retirement Survey, which reported that involuntary job loss increased the risks for stroke 2.64-fold, but increases the risk for MI by 1.89-fold [6]. From a life course perspective, job loss may be an exceptionally stressful experience, which can provoke undesirable health outcomes that include stroke and cardiovascular disease. The effects of major life events on MI and stroke were explored in the Copenhagen City Heart Study [35]. The results suggested that major life events in adults are associated with a stroke risk with a maximum HR of 1.60 (95% CI = 1.12–2.30), whereas the HR for MI is only 1.14 (95% CI = 0.73–1.78). In this relationship, adjustment for vital exhaustion attenuated the risk for stroke by approximately 30%. The authors suggested that the increased risk for stroke associated with major life events was partly explained by vital exhaustion, which could be mediated through psychosocial factors.

The results of this study should be interpreted within the context of its limitations. First, the incidences of stroke and cardiovascular disease were measured using self-reported questionnaires. However, we conclude that assessment of diagnosed disease using self-report is apparently not a source of major bias in our study because we examined significant life events, which are less likely to be misreported by the victims. A second limitation is that with the exception of age, gender, smoking history, BMI, exercise habit, and alcohol habit, we could not adjust for other risk factors for stroke or cardiovascular disease (e.g., family history, high-salt diet) because of data limitations. Third, because of the uneven distributions of age and gender and the low incident rates of stroke and cardiovascular disease, we had insufficient statistical power to investigate whether the risks of cardiovascular disease and stroke caused by unemployment were disproportionately present in certain socio-demographic subgroups. The problem of insufficient power was apparent in the gender stratification analysis. The estimated risk among female subjects was substantial in magnitude but statistically insignificant. Fourth, because those who experienced employment change during the follow-up period were excluded, subjects who left their job due to a health problem (including stroke or cardiovascular disease) could be excluded from the analysis. In addition, because we excluded those who had existing cerebrovascular or cardiovascular disease, there is a possibility that subjects who experienced heart problems immediately after job loss were excluded from the analysis. This could have led to over- or underestimation of risk. Moreover, the different distributions of employment status between respondents and non-respondents in the surveys could be a potential source of bias.

The present study did have some important strengths. First, it assessed a representative sample of the general Korean population. Second, to our knowledge, our study is the first to investigate the health effects of involuntary job loss in Asian countries. Third, our examination of effects modification by gender and the nature of job loss and different effects on development of stroke or cardiovascular disease may have provided a better opportunity to understand mechanisms and develop practical prevention strategy.

In conclusion, both voluntary retirement and involuntary job loss increased the risk for stroke or cardiovascular disease in middle-aged to older males. The risk associated with involuntary job loss was higher than the risk associated with voluntary retirement. Both voluntary retirement and involuntary job loss are more strongly related to the development of stroke than cardiovascular disease. We hope that these findings contribute to the establishment of a comprehensive public policy agenda aimed at promoting healthy retirement. Recognition of these patterns is important for policy makers so they can design health maintenance programs for this potentially vulnerable group. On the other hand, physicians who treat individuals who experiencing or experienced late career job loss also should pay more attention and consider their loss of employment as a risk factor for adverse vascular health outcomes, especially for stroke after male worker's involuntary job loss.

## References

[1] Yeung WJ, Hofferth SL (1998) Family adaptations to income and job loss in the US. Journal of Family and Economic Issues 19: 255–283.

[2] Stephens Jr M (2004) Job loss expectations, realizations, and household consumption behavior. Review of Economics and statistics 86: 253–269.

[3] Ensminger ME, Celentano DD (1988) Unemployment and psychiatric distress: social resources and coping. Soc Sci Med 27: 239–247. 3175706 10.1016/0277-9536(88)90127-x

[4] van Rijn RM, Robroek SJ, Brouwer S, Burdorf A (2013) Influence of poor health on exit from paid employment: a systematic review. Occupational and environmental medicine: oemed-2013–101591 10.1136/oemed-2013-101591 24169931

[5] Falba T, Teng HM, Sindelar JL, Gallo WT (2005) The effect of involuntary job loss on smoking intensity and relapse. Addiction 100: 1330–1339. 16128722 10.1111/j.1360-0443.2005.01150.x PMC1351253

[6] Gallo WT, Bradley EH, Falba TA, Dubin JA, Cramer LD, et al (2004) Involuntary job loss as a risk factor for subsequent myocardial infarction and stroke: findings from the Health and Retirement Survey. American journal of industrial medicine 45: 408–416. 15095423 10.1002/ajim.20004 PMC1351254

[7] Gallo WT, Bradley EH, Siegel M, Kasl SV (2000) Health effects of involuntary job loss among older workers findings from the health and retirement survey. The Journals of Gerontology Series B: Psychological Sciences and Social Sciences 55: S131–S140. 10.1093/geronb/55.3.s131 11833981

[8] Gallo WT, Bradley EH, Siegel M, Kasl SV (2001) The Impact of Involuntary Job Loss on Subsequent Alcohol Consumption by Older Workers Findings From the Health and Retirement Survey. The Journals of Gerontology Series B: Psychological Sciences and Social Sciences 56: S3–S9. 10.1093/geronb/56.1.s3 11192343

[9] Mandal B, Roe B (2007) Job loss, retirement and the mental health of older Americans. Retirement and the Mental Health of Older Americans (June 2007) 19096091

[10] Marshall VW, Clarke PJ, Ballantyne PJ (2001) Instability in the Retirement Transition Effects on Health and Well-Being in a Canadian Study. Research on aging 23: 379–409.

[11] Salokangas R, Joukamaa M (1991) Physical and mental health changes in retirement age. Psychotherapy and psychosomatics 55: 100–107. 1891555 10.1159/000288415

[12] Bunn AR (1979) Ischaemic heart disease mortality and the business cycle in Australia. American Journal of public health 69: 772–781. 453409 10.2105/ajph.69.8.772 PMC1619240

[13] Franks PJ, Adamson C, Bulpitt PF, Bulpitt CJ (1991) Stroke death and unemployment in London. Journal of epidemiology and community health 45: 16–18. 2045738 10.1136/jech.45.1.16 PMC1060695

[14] Brackbill RM, Siegel PZ, Ackermann SP (1995) Self reported hypertension among unemployed people in the United States. Bmj 310: 568. 7888932 10.1136/bmj.310.6979.568 PMC2548941

[15] Moon JR, Glymour MM, Subramanian S, Avendaño M, Kawachi I (2012) Transition to retirement and risk of cardiovascular disease: Prospective analysis of the US health and retirement study. Social Science & Medicine 75: 526–530. 22607954 10.1016/j.socscimed.2012.04.004 PMC3367095

[16] Olesen K, Rugulies R, Rod NH, Bonde JP (2014) Does retirement reduce the risk of myocardial infarction? A prospective registry linkage study of 617 511 Danish workers. International journal of epidemiology 43: 160–167. 24408969 10.1093/ije/dyt260

[17] Dupre ME, George LK, Liu G, Peterson ED (2012) The cumulative effect of unemployment on risks for acute myocardial infarction. Archives of internal medicine 172: 1731–1737. 23401888 10.1001/2013.jamainternmed.447

[18] Lundin A, Falkstedt D, Lundberg I, Hemmingsson T (2014) Unemployment and coronary heart disease among middle-aged men in Sweden: 39 243 men followed for 8 years. Occupational and environmental medicine: oemed-2013–101721 10.1136/oemed-2013-101721 24401871

[19] Browning M, Heinesen E (2012) Effect of job loss due to plant closure on mortality and hospitalization. Journal of Health Economics 31: 599–616. 22664774 10.1016/j.jhealeco.2012.03.001

[20] Garcy AM, Vågerö D (2012) The length of unemployment predicts mortality, differently in men and women, and by cause of death: A six year mortality follow-up of the Swedish 1992–1996 recession. Social Science & Medicine 74: 1911–1920. 22465382 10.1016/j.socscimed.2012.01.034

[21] WHO/IASO/IOTF (2000) The Asia-Pacific perspective: redefining obesity and its treatment. Melbourne

[22] Gallo WT, Bradley EH, Teng H-M, Kasl SV (2006) The effect of recurrent involuntary job loss on the depressive symptoms of older US workers. International archives of occupational and environmental health 80: 109–116. 16710713 10.1007/s00420-006-0108-5 PMC1904500

[23] Westerlund H, Vahtera J, Ferrie JE, Singh-Manoux A, Pentti J, et al (2010) Effect of retirement on major chronic conditions and fatigue: French GAZEL occupational cohort study. Bmj 341 10.1136/bmj.c6149 PMC2990862 21098617

[24] OECD (Organisation for Economic Co-operation and Development).StatExtracts Labror force statistics by ses and age [internet]. Paris: OECD Available from: http://stats.oecd.org/Index. aspx?DatasetCode = AVE_HRS.

[25] Statistics Korea. Statistics for the elderly, 2011 [cited 2014 May 1]. Available from: http://kosis.kr/ups/ups_01List01. jsp?pubcode = KO (Korean).

[26] Park S, Cho S-I, Jang S-N (2012) Health Conditions Sensitive to Retirement and Job Loss Among Korean Middle-aged and Older Adults. Journal of Preventive Medicine and Public Health 45: 188–195. 22712046 10.3961/jpmph.2012.45.3.188 PMC3374969

[27] Eliason M, Storrie D (2009) Job loss is bad for your health–Swedish evidence on cause-specific hospitalization following involuntary job loss. Social Science & Medicine 68: 1396–1406. 19243870 10.1016/j.socscimed.2009.01.021

[28] Ekerdt DJ, Kosloski K, DeViney S (2000) The normative anticipation of retirement by older workers. Research on aging 22: 3–22.

[29] Buxton JW, Singleton N, Melzer D (2005) The mental health of early retirees. Social Psychiatry and Psychiatric Epidemiology 40: 99–105. 15685400 10.1007/s00127-005-0866-5

[30] Iversen L, Klausen H (1986) Alcohol consumption among laid-off workers before and after closure of a Danish ship-yard: A 2-year follow-up study. Social Science & Medicine 22: 107–109. 3952525 10.1016/0277-9536(86)90314-x

[31] Bolton KL, Rodriguez E (2009) Smoking, drinking and body weight after re-employment: does unemployment experience and compensation make a difference? BMC Public Health 9: 77. 19267893 10.1186/1471-2458-9-77 PMC2678120

[32] Kim JE, Moen P (2002) Retirement Transitions, Gender, and Psychological Well-Being A Life-Course, Ecological Model. The Journals of Gerontology Series B: Psychological Sciences and Social Sciences 57: P212–P222. 10.1093/geronb/57.3.p212 11983732

[33] Broman CL, Hamilton VL, Hoffman WS, Mavaddat R (1995) Race, gender, and the response to stress: Autoworkers' vulnerability to long-term unemployment. American Journal of Community Psychology 23: 813–842. 8638552 10.1007/BF02507017

[34] Artazcoz L, Benach J, Borrell C, Cortès I (2004) Unemployment and mental health: understanding the interactions among gender, family roles, and social class. American Journal of public health 94: 82. 14713703 10.2105/ajph.94.1.82 PMC1449831

[35] Kornerup H, Osler M, Boysen G, Barefoot J, Schnohr P, et al (2010) Major life events increase the risk of stroke but not of myocardial infarction: results from the Copenhagen City Heart Study. European Journal of Cardiovascular Prevention & Rehabilitation 17: 113–118. 20038841 10.1097/HJR.0b013e3283359c18 PMC3634577

